# General rather than specific: Cognitive deficits in suppressing task irrelevant stimuli are associated with buying-shopping disorder

**DOI:** 10.1371/journal.pone.0237093

**Published:** 2020-08-04

**Authors:** Nico Lindheimer, Jennifer Nicolai, Morten Moshagen

**Affiliations:** 1 Department of Psychiatry and Psychotherapy, Charité - Universitätsmedizin, Berlin, Germany; 2 Department of Clinical Psychology and Psychotherapy, Ulm University, Ulm, Germany; 3 Psychological Research Methods, Department of Psychology, Ulm University, Ulm, Germany; University of Queensland, AUSTRALIA

## Abstract

**Objective:**

To investigate associations between buying-shopping disorder (BSD) propensity and the performance in the Stroop Matching Task. This task measures stimulus interference, one specific component of behavioral impulsivity, using neutral (i.e. not buying related) stimuli. Deficits thus mirror a general rather than a specific deficit to resist task-irrelevant stimuli.

**Method:**

222 participants completed the Stroop Matching Task, the Pathological Buying Screener, and various questionnaires assessing clinical background variables as well as trait-impulsivity.

**Results:**

Correlation analyses showed that BSD propensity was associated with poorer performance in the Stroop Matching Task. Multiple regression analyses controlling for related disorders and trait-impulsivity indicated that BSD was the only significant predictor.

**Conclusion:**

These findings indicate that BSD propensity is associated with deficits in the stimulus interference component of behavioral impulsivity, mirroring a general cognitive deficit. Since no other disorder significantly predicted the performance in the Stroop Matching Task, this deficit seems to be unique for BSD.

## Introduction

*Buying-shopping disorder (BSD)*, also referred to in the literature as compulsive buying, compulsive shopping, or pathological buying, is characterized as the inability to control recurring and overpowering buying impulses. The realization of buying impulses in excessive buying leads to substantial distress and negative consequences [1]. Proposed diagnostic criteria include: (1) frequent preoccupation with buying or impulses to buy that are experienced as irresistible; (2) frequent uncontrollable buying behaviors, including buying of more than can be afforded, buying of items that are not needed, or shopping periods that are longer than intended; (3) the excessive buying continues despite adverse consequences [2, 3]. This characterization implies that both cognitive and behavioral factors contribute to the acquisition, development, and maintenance of BSD. The prevalence of BSD in the general adult population was estimated at approximately 5% [4]. BSD is not included in the *American Psychiatric Association’s Diagnostic and Statistical Manual of Mental Disorders* (DSM-5). In the *International Classification of Diseases*, version 11 (ICD-11), BSD can be classified in the category “other specified impulse control disorders”.

Given its phenomenology, BSD has often been linked to impaired impulse and interference control [5]. Empirical evidence has shown that the broad construct of impulsivity has to be divided into two subcomponents that show little overlap: Dispositional or trait impulsivity, which is assessed by self-report measures, and behavioral or state impulsivity, assessed by experimental tasks [6]. Most studies on the role of impulsivity in BSD have relied on self-report measures. Among the different facets of trait impulsivity, *negative urgency*, defined as the tendency to commit rash or regrettable actions in response to negative affect, was consistently found to be associated with BSD [5, 7–11].

Whereas the use of self-report measures of impulsivity has some advantages (e.g., cost-effectiveness, ease of administration), it also has notable limitations including the limited possibility of mapping of underlying cognitive processes [6]. Yet, assessing the specific cognitive processes involved in BSD might be of particular importance for a better understanding of the disorder [12]. A valid option to assess such processes occurring within individuals are tasks measuring behavioral or state impulsivity [6]. It is generally assumed that (lack of) impulse control can be evident at different steps during goal selection and goal pursuit, with each step in the selection-action cycle being associated with different cognitive processes [6, 13]. Selecting an adequate action plan may require resisting short-term rewards in order to perform a behavior that is associated with desirable consequences in the long term (*delay discounting)*. Individuals also differ with respect to the amount of cognitive elaboration prior to deciding about when and how to act (*information sampling*). Having selected an action plan, pursuing the goal-directed behavior may be interfered in several ways, so that higher impulsivity is evident in difficulties to resist or to control interferences. Interference may occur by distracting stimuli in the environment (*stimulus interference*), by conflicting stimuli representations in memory (*proactive interference*), by concurrently activated conflicting response tendencies (*response interference*), or by difficulties to withhold or stop an ongoing response (*behavioral inhibition*). Finally, individuals differ in their perceptions of time, such that more impulsive individuals believe that time passes more slowly (*distortions in judging elapsed time*). Thus, given that behavioral impulsivity is considered a multifaceted construct [6, 13], it seems promising to examine how individual components of behavioral impulsivity are differentially associated with BSD.

To date, an increasing amount of studies can be identified with such an attempt. Derbyshire et al. [14] as well as Vogt et al. [10], used a version of the Stop-Signal Task to assess the ability to control or suppress an initiated or ongoing response (response inhibition) in individuals with BSD and healthy controls. Whereas Derbyshire et al. reported a significantly worse performance of individuals with BSD, Vogt et al. did not find significant differences between these groups. Nicolai, Darancó, and Moshagen [12] showed that BSD propensity was associated with impaired response inhibition as measured by the Go/No-Go task, in particular when the mood was negative. Furthermore, Nicolai and Moshagen [15] used two experimental tasks to assess the relationship between symptoms of BSD and delay discounting, the tendency to prefer smaller, immediate rewards to larger, but delayed rewards. They found that BSD was associated with higher levels of impulsivity as it predicted steeper discounting functions in both tasks over and above symptoms of comorbid disorders and trait impulsivity. The same authors could also show that BSD propensity was associated with difficulties in judging elapsed time, mirroring a general deficit in a specific component of state impulsivity [16]. Finally, several studies investigating decision making under risk and under ambiguity using several tasks (Game of Dice Task; Iowa Gambling Task, and the Cambridge Gambling Task) provide first indications of impulsivity in decision making and goal selection (which is related to the information sampling component of behavioral impulsivity [13]). However, these studies yielded mixed results for both, decision-making under ambiguity [8, 17, 18] and decision-making under risk [14, 17]. In tandem, these studies might indicate that decision-making seems to be impaired in BSD in situations of ambiguity, but not in situations where the decision outcome is explicit and stable [17].

Given that individuals with BSD report to be especially attracted by colors, sounds, lightning, and smells in retail stores as well as by the texture of clothing [19], the *stimulus interference* component of behavioral impulsivity seems to be of particular interest in BSD. However, no study has yet investigated this specific dimension of behavioral impulsivity in the context of BSD. Stimulus interference is defined as the ability to resist interference from task-irrelevant external environmental stimuli [13]. Individuals with BSD also report feeling more influenced by advertising than controls, especially via magazines, billboards, and Internet ads [20]. According to the cognitive-behavioral model proposed by Kellet and Bolton [21] a prototypical buying process could start like this: You sit at your computer and have to write a paper. You just want to do some quick research online. You do not find what you are looking for right away and you are a little frustrated. An online ad pops up that attracts your attention right away triggering an urge to shop. Eventually you decide to make some purchases online instead of finishing your paper. Two experimental studies provide further support for the role of external buying stimuli in triggering higher levels of arousal, subjective craving and urge to buy, and a higher skin-conductance response in individuals with BSD compared to controls [22, 23].

Taken together, the available evidence indicates difficulties to inhibit buying-related stimuli. However, it is currently unknown whether this impairment reflects a more general cognitive deficit to inhibit task-irrelevant stimuli that are unrelated to buying. Büttner et al. [24] argue for a domain-specific deficit on the basis of their finding that BSD propensity was associated with an attentional bias towards distracting products only when the task was framed as a shopping task. In contrast, Trotzke et al. [23] report higher physiological responses in individuals with BSD compared to healthy controls with respect to both buying and control cues, which could reflect a more general deficit. In light of these conflicting positions, the aim of the present study is to test the hypothesis that BSD propensity is associated with impaired performance in tasks measuring stimulus interference with neutral (i.e., buying-unrelated cues), which would mirror a general cognitive deficit to resist interference from task-irrelevant external stimuli.

## Methods

The study was approved by the ethics committee of the University of Mannheim, Germany (because two of the authors, NL and JN, were employed at the University of Mannheim at the time the study was performed). The study was conducted online in German language, closely adhering to standards of web-based experimenting [25].

### Participants

Participants were recruited via various social media networks. Furthermore, domain owners of self-help groups and online forums about BSD, obsessive-compulsive disorder (OCD), or multiple forms of addiction in Germany, Austria, and Switzerland were contacted and asked to present the study to their members. The data collection method complied with the terms and conditions for the websites from which the participants have been recruited. The rationale for this sampling procedure was that (1) individuals with BSD might misinterpret their symptoms as either an OCD or a form of addiction and therefore join these self-help groups and (2) individuals with BSD have been found to manifest high rates of comorbidities, including OCD and several forms of substance use disorders (e.g. alcohol use and smoking) and behavioral addictions (e.g. gambling disorder and compulsive internet use) [19, 26, 27]. In total, 225 participants completed the study. Three participants had to be excluded from the analysis due to indications of non-serious participation. The final sample consisted of 222 individuals (150 female, 69 male, 3 other). Mean age was 27.07 years (*SD* = 8.35; range 19–63). The majority of participants were students (71.62%), and another 22.52% of the participants were currently employed. Participants were not given any compensation for participation in the study. The study was completed on an anonymous and voluntary basis.

### Measures

The primary outcome was the performance in the Stroop Matching Task. This task was selected because it allows for a process-pure measurement of the stimulus interference component of behavioral impulsivity [13]. Self-report questionnaires used in this study include scales to assess BSD and several other clinical background variables that might be relevant in the context of BSD. Furthermore, a self-report measure of trait impulsivity was administered. Except for the ASSIST (see below), all questionnaire measures used 5-point Likert scales to assess symptom severity.

#### Stroop Matching Task

**Fig 1:**
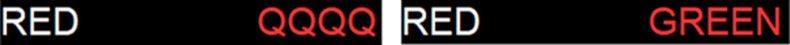
Two example stimulus displays of the Stroop Matching Task (not to scale). The task is to decide whether the probe stimulus (the left stimulus) matches the target stimulus (the right stimulus). In neutral match trials (displayed in the left panel), the target is a meaningless letter string (QQQQ) printed in the same color as the probe (red). In incongruent match trials (displayed in the right panel), the target stimulus names a color (GREEN) that is different from the printed color (red). The difference in performance between the incongruent and the neutral match trials is used to compute the Stoop Matching scores.

In each trial, the stimulus display comprised two strings of capital letters in the center of a black screen (see Fig 1). The probe always denoted one of four color names (the German words for “RED”, “YELLOW”, “GREEN”, and “BLUE”). The probe was presented at the left half of the screen in a neutral light gray. The target was presented on the right half of the screen in one of the four aforementioned colors. The task was to indicate whether the semantic meaning of the probe matched the color of the target or not by pressing the “i” key or the “e” key, respectively. In case of an incorrect response, a red cross appeared briefly in the center of the screen. The target stimulus could either be a meaningless string of consonants (“QQQQ”; neutral stimulus) or it denoted the name of one of the four colors. In the latter case, the meaning of the target could either match (congruent stimulus) or mismatch (incongruent stimulus) the target color. A combination of matching and congruency levels resulted in six different stimulus conditions. In this task, interference can arise when wrong stimulus features are compared. In order to introduce participants gradually to the task, a first training block of six trials with only neutral stimuli as targets was followed by another training block of twelve trials with color words as targets. This was followed by the test block comprising 120 trials in random order. The Stroop Matching Task is specifically recommended for the process-pure measurement of stimulus interference due to its response mechanism [13]. In contrast, in the classic Stroop task that has been used in previous research, participants do not merely indicate whether probe and target match or mismatch, but they are required to name the color of the target [28]. Thus, in this task, stimulus and response dimensions overlap, meaning that the classic Stroop interference effect involves both stimulus and response interference components, which makes this task not well suited to assess stimulus interference.

#### Pathological Buying Screener (PBS)

The PBS [29] is a screening instrument for BSD, consisting of 13 items. The internal consistency of the total PBS score can be considered excellent. Convergent validity has been demonstrated with the Compulsive Buying Scale [30].

#### Obsessive Compulsive Inventory (OCI-R)

The OCI-R [31] is a self-report measure for assessing symptoms of obsessive-compulsive disorder (OCD) with 18 items. The OCI-R differentiates well between individuals with and without OCD, internal consistency and test-retest reliability can be considered good to excellent, and evidence for both convergent and discriminant validity has been provided [31].

#### Behavioral Addiction Scale (BAS)

The BAS is an ad-hoc scale based on the Exercise Addiction Inventory (EAI; [32]). In its original form, the EAI is a self-report measure consisting of six items assessing exercise addiction. The scale was modified and extended by using the components of behavioral addiction suggested by [33]. In the first step, respondents choose among several activities (computer gaming, exercising, gambling, sex, and working) the one they prefer or pursue the most. In the next step, the items of the EAI have to be answered for the chosen behavior. In its original form, the EAI demonstrates very good internal consistency, content validity, concurrent validity, and construct validity [32]. The questionnaire can be retrieved in German and English from https://osf.io/fgcvs/.

#### The Alcohol, Smoking and Substance Involvement Screening Test (ASSIST)

The ASSIST [34] is an eight-item screening instrument designed to assess the use of psychoactive substances (alcohol, cannabis, cocaine, amphetamines, inhalants, sedatives, hallucinogens, and opiates) and related problems. The risk scores for each substance group range from 0 to 39, with values above 3 (above 10 for alcohol) indicating a moderate risk and values above 26 indicating a high risk of problems related to substance use. The ASSIST has demonstrated substantial test-retest reliability for each question and drug category and good internal consistency for the majority of domains [35]. Furthermore, evidence for convergent, construct, discriminative, and predictive validity has been provided [35]. In the present study, only the alcohol scale of the ASSIST was considered, because most participants indicated to have no experience with the remaining substances.

#### Borderline Symptom List 23 (BSL-23)

The BSL-23 [36], a 23 item self-report measure, was used to assess symptoms specific for borderline personality disorder. The BSL-23 has demonstrated very good internal consistency and evidence for convergent validity has been provided [36]. Furthermore, it discriminates well between patients with borderline personality disorder and other patients with different Axis I disorders.

#### Altman Self-Rating Mania Scale (ASRM)

The ASRM [37] is a self-report scale with five items for the assessment of the presence and/or severity of manic symptoms in the last seven days. The ASRM demonstrated good test-retest reliability. Adequate internal consistency and evidence for concurrent validity have also been provided [37].

#### Impulsive Behavior Scale (I-8)

The I-8 [38] is an eight-item-scale for the assessment of trait impulsivity. The scale covers four dimensions: (lack of) perseverance, (lack of) premeditation, sensation seeking, and urgency following the conceptualization of trait impulsivity by [39]. Test-retest reliabilities for the four dimensions range between .46 and .57 and McDonald’s Omega, a parameter similar to Cronbach’s α, ranges between .65 and .92. Evidence for convergent, discriminant and predictive validity has been presented [38].

### Procedure

The study took approximately 15 minutes to complete. After providing informed consent, demographic background data were collected, and a color blindness test was administered with an Ishihara plate, to detect and exclude participants with impaired red-green color vision from the study [40]. A detailed description of the task followed, and participants were instructed to respond as fast and as accurate as possible. After completing the Stroop Matching Task, the self-report questionnaires were presented in random order. Finally, participants were thanked for their participation, debriefed, and were given the contact information of the researchers to request further information.

### Analyses

Data were collected and analyzed anonymously. The primary outcome was the control of stimulus interference as measured by the performance in the Stroop Matching Task. In this task, a higher level of stimulus interference can be evident in higher error rates and/or longer response times. Since the error rate can be reduced by strategically slowing down responses (and vice versa), task performance was computed by taking the mean of the z-standardized errors and response times (as suggested by Stahl et al. [13]). The Stroop Matching score was then computed as the difference between incongruent match and neutral match conditions (comprising 24 trails each), such that higher values indicate more stimulus interference [13]. To facilitate the interpretation of the results, separate scores were additionally computed for response times (Stroop RT) and error rates (Stroop Accuracy). Prior to the analyses, all trials were excluded for which response times were either below 200 milliseconds, more than three interquartile ranges below the first quartile, or more than three interquartile ranges above the third quartile of an individual’s response time distribution for the respective task [13]. Associations between the PBS and the remaining measures with performance in the Stroop Matching Task were assessed via Pearson correlations. In addition, multiple linear regression analyses were performed on the Stroop performance to control for covariates. All analyses were performed in SPSS version 22.0. The alpha-error level was set to 5%.

## Results

The data of the current study can be retrieved from the open science framework at https://osf.io/fgcvs/. Detailed information about the descriptive statistics, the correlations between the questionnaire measures, as well as the Cronbach’s alphas are provided in Table 1. According to the guidelines proposed by Mueller et al. [29], 16.67% of the sample could be classified as individuals with BSD using the cutoff score of 29 (corresponding to two standard deviations above the mean in a general population sample). The PBS exhibited moderately positive correlations with the OCI-R (*r* = .34) and the BSL-23 (*r* = .45) and weakly positive correlations with the BAS (*r* = .19) (Table 1). Furthermore, significant weak to moderate correlations were evident between the PBS and the I-8 scales perseverance (*r* = -.27), sensation seeking (*r* = -.15), and negative urgency (*r =* .32).

**Table 1:** Means, standard deviations, and correlations (Cronbach’s α estimate of internal consistency on the diagonal).

		Correlations												
Variable	Mean (SD)	1	2	3	4	5	6	7	8	9	10	11	12	13
1. PBS	22.36 (8.40)	(.93)												
2. OCI-R	14.56 (10.31)	.34**	(.89)											
3. ASSIST Alc.	7.79 (6.51)	.06	.00	(.73)										
4. BAS	15.55 (4.00)	.19**	.21**	.08	(.68)									
5. BSL-23	34.44 (13.92)	.45**	.54**	-.04	.13	(.96)								
6. ASRM	7.82 (3.62)	-.05	-.02	.24**	.01	-.30**	(.77)							
7. I-8 Urgency	3.03 (0.79)	.32**	.11	.20**	.11	.11	.12	(.63)						
8. I-8 Premeditation	3.33 (0.61)	.12	.05	.21**	.06	.00	.01	.23**	(.82)					
9. I-8 Perseverance	3.56 (0.84)	-27**	-.05	-.09	-.04	-.21**	.09	-.29**	-.01	(.57)				
10. I-8 Sensation Seeking	3.42 (0.56)	-.15*	-.10	.07	.07	-.22**	.07	.24**	.07	.32**	(.86)			
11. Stroop Matching	0.00 (0.78)	.21**	.02	-.10	.08	-.04	-.08	.04	-.03	-.01	-.04	-		
12. Stroop RT	194.04 (202.20)	.27**	.02	-.11	.07	.04	-.06	.05	-.03	.01	-.05	.78**	-	
13. Stroop Accuracy	0.07 (0.77)	.06	.01	-.05	.04	.02	-.07	.01	-.02	-.02	-.01	.78**	.21**	-

*N* = 222. PBS = Pathological Buying Screener, OCI-R = Obsessive-Compulsive Inventory, ASSIT Alc. = Alcohol Scale of The Alcohol, Smoking and Substance Involvement Screening Test, BAS = Behavioral Addiction Scale, BSL-23 = Borderline Symptom List 23, ASRM = Altman Self-Rating Mania Scale, I-8 = Impulsive Behavior Scale (I-8) Stroop Matching = Stroop Matching score, Stroop RT = response time in the Stroop Matching Task, Stroop Accuracy = error rate in the Stroop Matching Task.

*p < .05.

**p < .01.

The PBS was the only variable that was significantly associated with impaired performance in the Stroop Matching Task (*r* = .21; note that higher values for the Stroop Matching score indicate more interference and thus less interference control). Follow-up analyses indicated that this performance deficit can be traced back to slower response times (*r* = .27), rather than to lower accuracy (*r* = .06, *p* = .185). Thus, interference in the Stroop Matching Task was evident in the requirement to slow-down responding in order to yield the same accuracy. Notably, in accordance with previous research concerning behavioral and trait impulsivity, none of the I-8 scales was significantly related to the stimulus interference measures.

To investigate whether the association between BSD and performance in the Stroop Matching Task holds when controlling for the remaining variables, multiple linear regression analyses were performed (Table 2). In the first step, the OCI-R, BAS, ASRM, BSL-23, and the Alcohol scale of the ASSIST were entered as background variables. This model did not account for a significant proportion of the variance in the Stroop Matching score (*R*^2^ = .02), nor did any of the predictors reach significance. In the second step, the scales of the I-8 were added, however, none of the scales predicted the Stroop Matching score significantly. In the final step, the PBS was included, which led to a significant increase in the variance explained (Δ*R*^2^ = .05, *p* < .01). The PBS score was the only significant predictor (*β* = 0.26, *p* < .01) in this model. However, the proportion of explained variance by the full model did not deviate significantly from zero (*R*^2^ = .08, *p* = .08) as a result of the many non-significant predictors included in the first steps.

**Table 2:** Multiple regression predicting the stroop matching score.

*Variable*	*β*	*t*	*p*	ΔR^2^	*F*	*p*
Step 1				.02	0.89	.49
ASRM	-.06	-0.76	.45			
OCI-R	-.10	-0.12	.90			
BSL-23	.01	0.16	.87			
BAS	.08	1.21	.23			
ASSIST Alc.	-.09	-1.31	.19			
Step 2				.01	0.48	.75
I-8 Urgency	.10	1.25	.21			
I-8 Premeditation	-.04	-0.49	.63			
I-8 Perseverance	.05	-0.59	.56			
I-8 Sensation Seeking	-.08	-0.97	.33			
Step 3				.05	10.68	< .01
PBS	.26	-0.27	< .01			
Total R^2^				.08	1.73	.08

*N* = 222. Standardized regression coefficients (β) with t-tests and p-values and increase in the proportion of variance explained (ΔR2) with F-tests and p-values. PBS = Pathological Buying Screener, OCI-R = Obsessive-Compulsive Inventory, ASSIST Alc. = Alcohol Scale of The Alcohol, Smoking and Substance Involvement Screening Test, BAS = Behavioral Addiction Scale, BSL-23 = Borderline Symptom List 23, ASRM = Altman Self-Rating Mania Scale, I-8 = Impulsive Behavior Scale (I-8).

Finally, the PBS variable was dichotomized at the cutoff point and group means of the Stroop Matching score were compared via a t-test. Results revealed that participants over the cutoff performed significantly worse than participants below the cutoff, *t*(41.71) = -1.93, *p* = .031. This difference corresponds to a medium effect according to Cohen (*d* = 0.47; 95% CI: 0.12–0.83).

## Discussion

BSD has been repeatedly associated with higher levels of impulsivity. However, this evidence mainly stems from studies that used self-report measures of trait impulsivity, while research on specific behavioral components of impulsivity assessed by behavioral tasks is scarce. To address this issue, the present study investigated stimulus interference as a specific component of behavioral impulsivity using the Stroop Matching Task. In particular, it was hypothesized that BSD propensity is associated with a general (rather than domain-specific) cognitive deficit in inhibiting task-irrelevant stimuli. Corroborating this hypothesis, results indicated that BSD propensity was associated with impaired stimulus interference control as measured by the performance in the Stroop Matching Task. Comparing participants scoring below and above the PBS cutoff score, respectively, indicated that this impairment amounts to an effect of medium size (*d* = .47). Given that related symptom measures and trait impulsivity were controlled in the analyses, BSD symptoms seem to be uniquely associated with deficits in the stimulus interference component of behavioral impulsivity. As such, this study is the first to provide direct evidence for the attribution of difficulties associated with BSD to resist external buying stimuli to a general rather than a domain-specific cognitive deficit. The general and condition-specific cognitive deficit might play a role in the acquisition, development, and maintenance of BSD and should, therefore, be subject to further research. A general deficit to ignore any distracting stimuli in the environment might be mirrored in an even more pronounced specific deficit to resist buying-related stimuli as observed by Büttner and colleagues [24], yet, due to a lack of longitudinal studies, experimental designs, and studies on causal relationships, it is currently only possible to speculate on the potential underlying mechanisms.

In line with previous research, little overlap was found between self-report measures and behavioral tasks assessing impulsivity [6]. This finding is consistent with the assumption that self-reports of trait impulsivity and laboratory-based behavioral tasks of state impulsivity tap different aspects of impulsivity [6]. However, both measures yielded significant associations with BSD. This suggests that each measure can independently explain variance in BSD. In line with this reasoning, a post-hoc regression analyses with the PBS as dependent variable yielded significant associations with the Stroop Matching score (β = 0.19, p < .01), urgency (β = 0.30, p < .01), and sensation seeking (β = -0.18, p < .01). Therefore, the inclusion of both self-reports and behavioral tasks of impulsivity seems to be essential in order to provide a comprehensive understanding of the factors that influence BSD. Due to the lack of research on behavioral impulsivity and BSD, it seems promising to intensify research efforts in this direction in future studies. However, it should be noted that the I-8-scales used to assess trait-impulsivity consist of only two items each, which could possibly be a weakness.

The present findings could also be useful for treatment approaches for individuals suffering from BSD. In particular, therapeutic interventions that focus on training stimulus control techniques might be effective for the treatment of the disorder. As such, the results of this study help to explain the beneficial effects of cognitive-behavioral group therapy on BSD, which have been found across three controlled psychotherapy studies that addressed motivation, stimulus control techniques, developing alternative behaviors, cognitive restructuring, exposure and response prevention techniques [41–43]. The results of the present study indicate a general deficit of individuals with BSD in dealing with stimulus interference. Translated into practice, the results imply that training of stimulus control strategies should not be limited to buying situations, but should also be aimed at general situations where individuals with BSD are at risk of being distracted by irrelevant stimuli that are unrelated to the current goal they are pursuing.

Some limitations have to be considered when interpreting the results of the present study. The results can only partly be generalized to clinical populations, as we used a sample recruited from online forums, self-help groups, and social media. However, since most researchers suggest a dimensional rather than categorical approach with symptoms of BSD ranging from very mild to severe (e.g. [2, 5, 10, 44]) , analog samples can be considered appropriate for the investigation of the underlying mechanisms of BSD, and the results are relevant for understanding its phenomena across the continuum of symptom severity [45]. Nevertheless, it should be examined whether the present findings can be replicated in patients seeking psychotherapeutic treatment for BSD. Similarly, it could be argued that the high percentage of students in the sample might have led to an underestimation of BSD since they often can rely on their parents when purchases exceed their monetary means and thus do not have to worry so much about debts as those employed. Yet, meta-analytic findings show that the prevalence of BSD is higher in students than in the general population [4]. A further limitation may result from the fact that depression and anxiety symptoms were not controlled in the present study, especially since such symptoms are very common in people with BSD and have been shown to affect performance in the classic Stroop task (e.g. [46]). Although this limitation should be considered when interpreting the present results, the observed effects are arguably not due to the confounding effects of depressive symptoms, because such a confound should also incur relations of borderline personality disorder [47] or problems related to alcohol use [48], given that both also show high comorbidities with affective disorders. Nevertheless, this weakness of the present study should be considered in future research. Moreover, we assessed the stimulus interference component of behavioral impulsivity using the Stroop Matching Task only. It would be interesting to investigate other tasks that also tap this component, in particular, the Shape- and Animal Matching tasks [13]. The fact that the vast majority of the sample identified as female might be considered a further limitation of the present study. However, most studies concerning BSD have a similar gender distribution with females being overrepresented [4]. Dittmar suspects that women are generally more affected by BSD than men, which might be related to traditional gender roles [49]. Still, future research could focus on recruiting a more balanced sample in this respect to target potential gender differences.

Despite the above-mentioned limitations, the present study demonstrates that BSD symptoms are uniquely associated with deficits in the stimulus interference component of behavioral impulsivity as measured by the Stroop Matching Task. As such, the results of the present study point to a general cognitive deficit to resist interference from task-irrelevant external stimuli.
